# Carnosic Acid Mediates Production of Reactive Oxygen Species to Regulate Mitogen‐Activated Protein Kinase Pathway Phosphorylation and Induce Apoptosis in Human Breast Cancer Cells

**DOI:** 10.1002/cam4.71446

**Published:** 2026-01-09

**Authors:** Xinyu Wang, Peng Xu, Sha Luan, Yuying Jiao, Yue Gao, Changjiu Zhao, Peng Fu

**Affiliations:** ^1^ Department of Nuclear Medicine The Fourth Affiliated Hospital of Harbin Medical University Harbin China; ^2^ Department of Nuclear Medicine The First Affiliated Hospital of Harbin Medical University Harbin China

**Keywords:** ^99m^Tc‐CN5DG, breast cancer, carnosic acid, ROS

## Abstract

**Background:**

Reactive oxygen species (ROS) can induce cancer cell apoptosis, which plays a crucial role in breast cancer therapy. Carnosic acid (CA) exerts an anti‐tumor effect via generating ROS or activating the mitochondria‐related apoptosis pathway in vitro and in vivo. The deoxy‐glucose derivative CN5DG labeled with ^99m^Tc can be used for breast cancer imaging and evaluating the therapeutic efficacy of CA.

**Methods:**

Inhibition of cancer cell proliferation by CA was assessed by MTT and cell colony‐formation assays. The mechanism of CA‐induced cancer cell apoptosis was examined by western blotting and the apoptosis rate was detected by flow cytometry. The in vivo anti‐tumor effect of CA was assessed by immunohistochemistry and ^99m^Tc‐CN5DG imaging. Its biodistribution was determined to evaluate the accumulation of ^99m^Tc‐CN5DG in xenografts.

**Results:**

CA promoted cancer cell apoptosis via ROS generation, which activated c‐Jun N‐terminal kinase (JNK) and p38 phosphorylation. The apoptosis rates of T47D and MCF7 cells treated with CA (IC50 concentration) for 24 h were 44.97% ± 1.56% and 39.74% ± 1.78%, respectively. The antioxidant N‐acetyl‐L‐cysteine (5 μM) abolished CA‐induced apoptosis. MCF‐7 xenografts without CA were visualized in vivo at all time points by ^99m^Tc‐CN5DG imaging (tumor/muscle ratio: 2.52 ± 0.10 at 4 h), but xenografts treated with CA were not visualized (tumor/muscle ratio: 1.36 ± 0.34 at 4 h). Evaluation of the biodistribution by γ‐counter also demonstrated the efficacy of ^99m^Tc‐CN5DG. Bcl‐2 and Ki‐67 expression were higher while Bax expression was lower in the control group compared with the CA treatment group. The tumor/muscle radioactivity count ratio was lower following treatment with CA compared with the control group.

**Conclusion:**

CA can induce breast cancer apoptosis via ROS generation and activation of JNK and p38 phosphorylation. The anti‐tumor effect of CA can be assessed using ^99m^Tc‐CN5DG SPECT imaging.

## 1 Introduction

The number of new breast cancer cases among women in the United States is anticipated to reach 310,720 by 2024, constituting 32% of all newly diagnosed female cancers, making it the leading cause of cancer among women [1]. Approximately 70% of breast cancer cases are hormone receptor‐positive, predominantly estrogen receptor (ER)‐positive [2]. Endocrine therapy is the mainstay of treatment for patients with hormone receptor‐positive breast cancer, mainly including selective ER modulators and downregulators and aromatase inhibitors. Despite treatment however, some patients develop endocrine‐therapy resistance and the exact mechanism of endocrine‐therapy resistance is not fully understood. Although the mammalian target of rapamycin (mTOR) inhibitor everolimus and the cyclin‐dependent kinase 4/6 inhibitor palbociclib have been used to prevent endocrine resistance in breast cancer [2], these drugs also have some side effects. We therefore aimed to identify a more effective, ER‐independent compound with fewer side effects, to overcome endocrine resistance in breast cancer.

**FIGURE 1:**
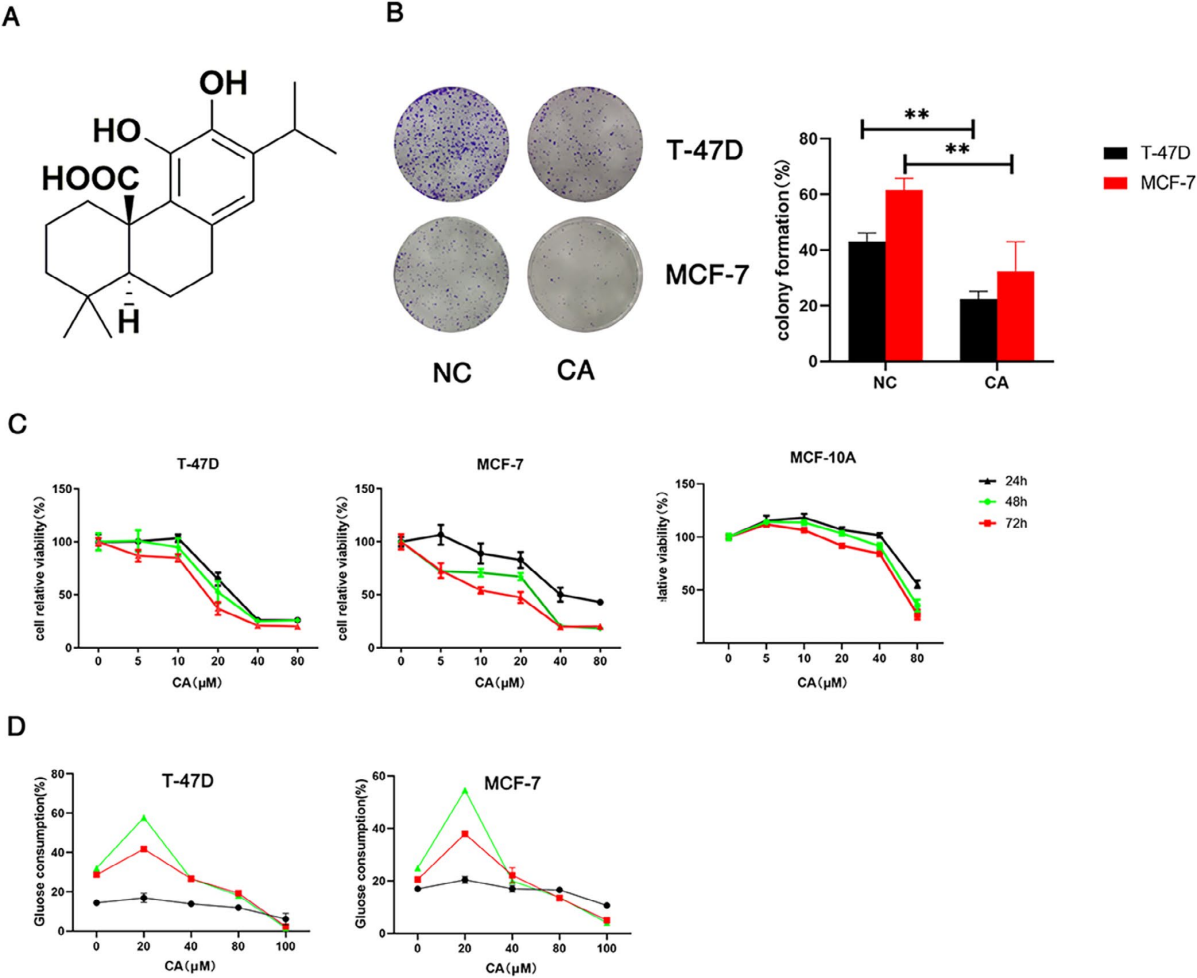
CA inhibited breast cancer cell growth and colony formation. (A) Chemical structural formula of CA. (B) T‐47D and MCF‐7 cells were treated with 10 μM CA for 6 h and the growth‐inhibition effects of CA were determined by colony‐formation assay. (C) MCF‐7, T‐47D, and MCF‐10A cells were treated with different concentrations of CA for 24, 48, and 72 h and their relative viabilities were evaluated by MTT assay. (D) T‐47D and MCF‐7 cells were treated with different concentrations of CA for 6, 12, and 24 h and the glucose consumption rates were examined using a glucose assay kit. Data presented as mean ± SD of three independent experiments. *p < 0.05, **p < 0.01 versus control group.

Carnosic acid (CA, Figure 1A) is a rosemary and sage extract [3] used as an antiseptic in products such as toothpaste, mouthwash, and chewing gum [4]. CA has demonstrated several functions, including antioxidant [5] and anti‐inflammatory activities [4], with no severe side effects. Increasing numbers of studies have shown that CA also has an excellent anti‐tumor effect. CA induced apoptosis of hepatocellular carcinoma cells via the reactive oxygen species (ROS)‐mediated mitochondrial pathway [6, 7, 8] and halted the progression of oral squamous cell carcinoma by inducing mitochondrial‐mediated apoptosis [9]. Furthermore, the combined application of CA and curcumin suppressed the proliferative activity and disrupted the mitochondrial function of metastatic prostate cancer cells compared with their individual uses [10]. CA has been discussed in various studies related to breast cancer, emphasizing several molecular pathways [11, 12]. In contrast, our research focused on the most effective pathway through which CA facilitated tumor apoptosis. This pathway enhanced hormone therapy and offered new insights for the treatment of breast cancer, including hormone‐resistant cases.

^18^F‐fluorodeoxyglucose (^18^F‐FDG) is a tumor‐imaging agent that releases positrons on decay, which can be captured by positron emission tomography (PET) during cancer imaging. ^18^F‐FDG is an important glucose derivative in tumor diagnosis, but its use is limited by its high cost. Technetium‐99 m (^99m^Tc) is a common radionuclide and an ideal low‐cost imaging agent for single photon emission computed tomography (SPECT) that can be obtained by elution from a molybdenum‐Tc generator. The ^99m^Tc‐labeled glucose derivative ^99m^Tc‐CN5DG is a promising imaging agent in lung cancer [13]. ^99m^Tc‐CN5DG offers multiple benefits for in vivo imaging. First, its preparation process is straightforward, requiring only 20 min compared with about 1 h for ^18^F‐FDG. Second, ^99m^Tc‐CN5DG exhibits higher tumor‐to‐muscle (T/M) and tumor‐to‐blood ratios compared with ^18^F‐FDG, leading to improved contrast in imaging studies [13]. Finally, the preparation of ^99m^Tc‐CN5DG is relatively inexpensive, costing about a quarter that of ^18^F‐FDG. ^99m^Tc‐CN5DG may thus be an effective imaging agent for tumor imaging and for evaluating the efficacy of chemotherapy.

In the present study, we investigated the mechanism of CA‐induced breast cancer apoptosis. We found that CA inhibited breast cancer proliferation and promoted breast cancer apoptosis via ROS generation, followed by activation of JNK and p38 phosphorylation. We determined the effects of CA on the expression levels of Bcl‐2, Ki‐67, and Bax in MCF‐7 xenografts, and also assessed its anti‐cancer effects using ^99m^Tc‐CN5DG.

## 2 Materials and Methods

### 2.1 Cell Lines and Culture

T‐47D, MCF‐7 (human breast cancer cell lines), and MCF‐10A (non‐tumorigenic human breast epithelial cell line) were purchased from the Chinese Academy of Sciences Shanghai Cell Bank. MCF‐7 and T‐47D cells were cultured in 1640 medium (Gibco) including 10% fetal bovine serum (PAN), penicillin (100 U/mL), and streptomycin (100 μg/mL). MCF‐10A cells were maintained in DMEM/F12 Basal Medium supplemented with 5% HS, 20 ng/mL epidermal growth factor, 0.5 μg/mL hydrocortisone, 10 μg/mL insulin, 1% NEAA, and 1% P/S (Procell). All cells were cultured in an incubator in 5% CO_2_ at 37°C.

### 2.2 Reagents

CA (> 98% purity) was purchased from J&K Chemical Ltd. (Beijing, China). BMS‐582949 and SP600125 were purchased from Selleck (Shanghai, China). N‐acetyl‐L‐cysteine (NAC) was purchased from Beyotime (Shanghai, China). 2′,7′‐Dichlorofluorescein diacetate (DCFH‐DA) was purchased from Sigma. CA, BMS‐582949, SP600125, and DCFH‐DA were dissolved in dimethyl sulfoxide (DMSO) and stored at –80°C. The concentration of DMSO in the experiments did not exceed 0.1% (v/v) in each group. NAC was dissolved in sterile phosphate‐buffered saline (PBS). The following antibodies were also used: rabbit monoclonal phospho (p)‐JNK, p‐p38 (Cell Signaling Technology, 9926 T), rabbit polyclonal β‐actin (Proteintech, 20,536–1‐AP), Bax (Proteintech, 50,599‐2‐Ig), Bcl‐2 (Proteintech, 12,789‐1‐AP), and poly‐ADP ribose polymerase (PARP, Cell Signaling Technology, 9542).

### 2.3 MTT Analysis

T‐47D, MCF‐7, and MCF‐10A (5 × 10^3^) cells were seeded in 96‐well plates and treated with CA (0–80 μm) for 24, 48, and 72 h at 37°C. Cell viability was then assessed by MTT assay (Beyotime). The relative viability was detected using a microplate reader (Tecan Infinite 200 Pro) and the absorbance was recorded at 570 nm. The half‐maximal inhibitory concentration (IC50) was calculated using GraphPad Prism 8.0 Professional analysis software.

### 2.4 Colony‐Forming Assay

T‐47D and MCF‐7 cells were treated with CA for 6 h at the IC50. Five hundred cells per well were cultured with complete medium in 6‐well plates for 10 days and then fixed with methyl alcohol for 5 min. The cells were washed three times with PBS and then stained with Giemsa (Solarbio) for 15 min. All colonies with > 50 cells were evaluated. The colony‐forming efficiency was calculated based on colonies/number of inoculated cells × 100%.

### 2.5 Flow Cytometric Analysis

T‐47D and MCF‐7 cells (3 × 10^5^ cells/well) were cultured in 6‐well plates and treated with CA at the indicated concentrations for 6, 12, and 24 h. The cells were collected in a centrifuge tube containing trypsin, and stained with annexin V and propidium iodide (PI) for 15 min, as described by the manufacturer (BD Biosciences, 556, 570).

### 2.6 Western Blotting

T‐47D and MCF‐7 cells were cultured in 6‐well plates (3 × 10^5^cells/well), washed with PBS after 24 h, and then treated with CA at the IC50 concentration (0–24 h). The medium was then discarded and the cells were treated with RIPA buffer; the mixture was collected and centrifuged (4°C, 14,000*g*, 15 min), and equivalent proteins were used for western blotting. The proteins were separated by 10% sodium dodecyl sulfate‐polyacrylamide gel electrophoresis and then transferred onto a polyvinylidene fluoride membrane (Thermo Fisher). Non‐specific binding sites were blocked for 2 h in TBST (containing 5% milk). All bands were incubated with primary antibodies overnight at 4°C. The following day, the bands were washed with TBST three times, incubated with secondary anti‐rabbit goat (1:4000) IgG (ZSGB‐Bio, ZB‐5301), and then combined with a chemiluminescence reagent (ECL Plus, Thermo Fisher). The chemiluminescence was observed using a Tanon 6200 luminescent imaging workstation.

### 2.7 Animal Xenograft Models

Female BALB/c nude mice (3–4 weeks old weighing 15–19 g) were implanted subcutaneously with MCF‐7 cells (1 × 10^7^) in the right shoulder. The mice were divided into two groups: a control group treated with 100 μL olive oil once every 2 days by oral gavage, and a treatment group treated with CA (50 mg/kg) dissolved in 100 μL olive oil twice a day by oral gavage. Tumor volume measured using Vernier calipers and body weight was measured every 7 days. After 4 weeks of treatment, mice in both groups underwent SPECT. All animal experiments were approved by the Harbin Medical University Animal Ethics Committee in accordance with Chinese legislation and followed relevant guidelines (approval number: No. 2017013). The institutional review board approved the experiments involving mice with breast cancer xenografts.

### 2.8 Radiosynthesis and Radiochemical Purity of ^99m^Tc‐CN5DG and ^99m^Tc‐MIBI

CN5DG was synthesized by Beijing Shihong Pharmaceutical Center (Beijing, China) and labeled with ^99m^Tc as described previously (9). Briefly, 1–3 mL ^99m^TcO_4_^−^ was added to a freeze‐dried CN5DG kit containing CN5DG (1 mg), SnCl_2_·2H_2_O (0.06 mg), sodium citrate (1 mg), and L‐cysteine (1 mg) and the mixture was then incubated for 20 min at 100°C. To determine the radiochemical purity of ^99m^Tc‐CN5DG, 1 μL of the sample was spotted on silica gel‐impregnated thin‐layer chromatography strips and the radioactivity distribution was determined using ammonium acetate (1 M): methanol (2:1) as the developing agent, using a thin‐layer chromatography scanner. The ^99m^Tc‐MIBI synthesis method is the same as above. The area under the peak ratio and the radiochemical purity were then calculated.

### 2.9 SPECT Imaging of Mouse Xenografts

SPECT imaging of xenografts was carried out using a clinical SPECT system (Discovery 670, GE Healthcare) equipped with a low‐energy high‐resolution pinhole collimator. All images were acquired with detector 1 for posterior static imaging at 0.5, 2, and 4 h, respectively. Image acquisition was terminated when the radioactivity count reached 100. The zoom was 1.00, the matrix was 128 × 128, and the energy peak was 140 keV. The images were analyzed using a Xeleris Functional Imaging Workstation.

### 2.10 Biodistribution of ^99m^Tc‐CN5DG and ^99m^Tc‐MIBI

The mice were divided into two groups (*n* = 12 per group). After 4 weeks of treatment, the mice were injected with ^99m^Tc‐CN5DG and ^99m^Tc‐MIBI (8.5 MBq) via the tail vein and then sacrificed (*n* = 4 per group) at 0.5, 2, and 4 h, respectively. The radioactivity was calculated using a gamma counter and tissue radioactivity was expressed as a percentage of injected dose per gram tissue (%ID/g).

### 2.11 Immunohistochemical Assays

Tumor, heart, liver, and kidney tissues were removed and fixed in 4% paraformaldehyde for 24 h. All tissues were embedded in paraffin, sectioned using a microtome, deparaffinized, and hydrated. Antigen repair was performed in PBS for 15 min at 92°C–98°C. The tumor slices were then blocked with bovine serum albumin (BSA), washed with PBS, and incubated with primary antibodies overnight at 4°C. The primary antibody (1:100) was dissolved in 5% BSA and PBS solution. Ki67 (Abcam, ab92742), Bcl‐2 (Abcam, ab32124), and Bax (Abcam, 32,503) were used to evaluate the effect of CA treatment. The following day, the tumor slices were incubated with biotin‐conjugated secondary antibodies. Finally, the tumor slices were stained using DAB solution and hematoxylin. Heart, liver, and kidney tissues were stained with hematoxylin–eosin (HE).

### 2.12 Statistical Analysis

Data are presented as the mean ± standard deviation (SD). Statistical analyzes were carried out using SPSS (version 20.0). The data were analyzed by one‐way analysis of variance and Student's *t*‐tests. *p* < 0.05 was considered statistically significant.

## 3 Results

### 3.1 CA Inhibited Breast Cancer Proliferation and Glucose Uptake

We evaluated the impact of CA on the proliferation of breast cancer and normal mammary epithelial cells by MTT assay. T‐47D and MCF‐7 cells were incubated with CA (0, 5, 10, 20, 40, 80 μM) for different times (24, 48, 72 h) (Figure 1C). CA significantly inhibited cell proliferation in time‐ and dose‐dependent manners. The IC50 values of T‐47D, MCF‐7, and MCF‐10A cells were 25.42, 20.93, and 69.63 μM at 48 h, respectively, indicating that breast cancer cells were more sensitive to CA than normal mammary epithelial cells. We also assessed the survival of T‐47D and MCF‐7 cell lines by colony‐formation assays (Figure 1B). Colony formation was much lower in the treated groups compared with the control groups in both T‐47D and MCF‐7 cells. We evaluated breast cancer cell metabolism by measuring the rate of glucose consumption in the culture medium (Figure 1D). Glucose consumption was accelerated by low concentrations of CA, but decreased with increasing time and CA concentration. These results indicated that CA significantly decreased the colony‐formation ability and metabolism of T‐47D and MCF‐7 cells.

### 3.2 CA Induced Breast Cancer Apoptosis

**FIGURE 2:**
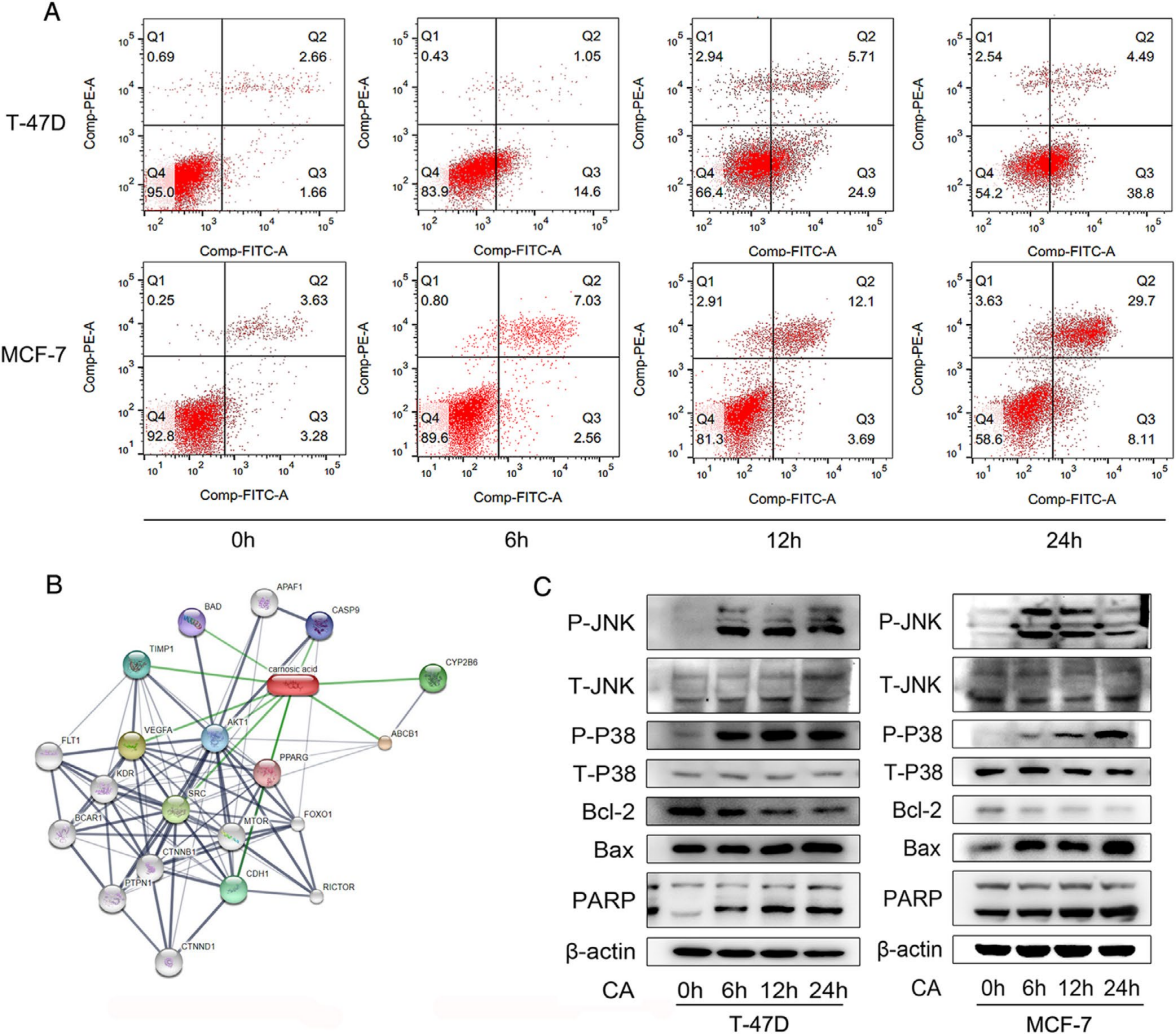
CA induced breast cancer cell apoptosis via p38 and JNK phosphorylation and regulated glucose metabolism. (A) T‐47D and MCF‐7 cells were treated with 25 μM and 20 μM CA for 6, 12, and 24 h, respectively. Cancer cell apoptosis was detected by flow cytometry using annexin V‐FITC/PI (B) Potential targets of CA. (C) Protein expression levels of p‐JNK, p‐p38, Bax, Bcl‐2, and PARP were measured by western blotting. Data presented as mean ± SD of three independent experiments. *p < 0.05, **p < 0.01 versus NC group.

To determine if CA induced apoptosis in breast cancer cells, we treated T‐47D and MCF‐7 cells with 25 and 20 μM CA, respectively, and measured the apoptosis rates by flow cytometry at different time points (Figure 2A). CA induced apoptosis in T‐47D and MCF‐7 cells after 6 h of treatment and the apoptosis rate peaked at 24 h (44.97% ± 1.56% and 39.74% ± 1.78%, respectively). Expression levels of Bax, Bcl‐2, and PARP were used as markers of tumor apoptosis. Bax and PARP expression levels increased significantly while Bcl‐2 expression decreased with time (Figure 2C). These results indicated that CA induced T‐47D and MCF‐7 cell apoptosis by up‐regulating Bax and PARP and down‐regulating Bcl‐2.

### 3.3 CA Activated JNK and p38 in Breast Cancer Cell Lines

**FIGURE 3:**
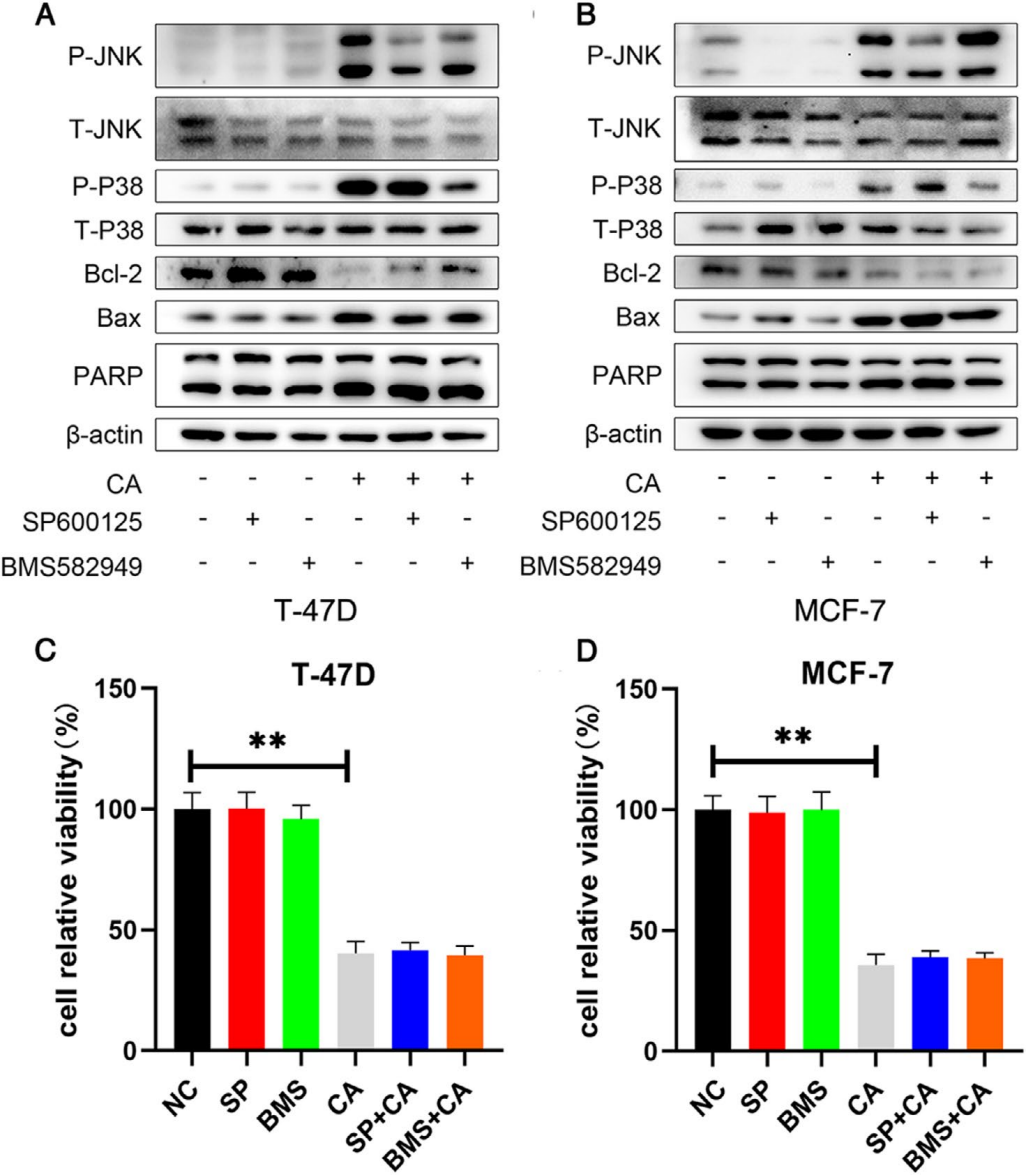
SP600125 and BMS582949 were used to inhibit CA‐induced JNK and p38 phosphorylation. (A) T‐47D and (B) MCF‐7 cells were pre‐treated with SP600125 and BMS582949 for 2 h, followed by treatment with CA at 25 μM and 20 μM, respectively. Protein expression levels of p‐JNK, p‐p38, Bax, Bcl‐2, and PARP were measured by western blotting. (C) and (D) Relative cell viability was evaluated by MTT assays. Data are presented as mean ± SD of three independent experiments. *p < 0.05, **p < 0.01 versus NC group.

To clarify the specific mechanism by which CA promoted breast cancer cell apoptosis, we predicted several targets and pathways using STITCH (Figure 2B). We examined the mTOR, signal transducer and activator of transcription (STAT) 3, Akt, and mitogen‐activated protein kinase (MAPK) signaling pathways by Western blotting. CA treatment had no effects on protein levels of mTOR, STAT3, and Akt (not shown), but CA induced p‐p38 and p‐JNK phosphorylation in T‐47D and MCF‐7 cells (Figure 2C). These results suggest that CA may promote breast cancer cell apoptosis by mediating p‐p38 and p‐JNK. T‐47D and MCF‐7 cells were then treated with CA with or without the JNK and p38 phosphorylation inhibitors SP600125 and BMS582949 (Figure 3A,B). Phosphorylation levels of JNK and p38 were repressed by CA in the breast cancer lines and by the phosphorylation inhibitors, but levels of cleaved PARP and Bax were not significantly decreased. Similar results were observed in MTT experiments (Figure 3C,D). SP600125 and BMS582949 did not rescue the CA‐induced suppression of cancer cell viability. These results indicated that JNK and p38 phosphorylation were inhibited but did not reduce tumor cell apoptosis, suggesting that other factors were responsible for regulating the apoptosis of tumor cells.

### 3.4 CA Induced Generation of ROS in Breast Cancer Cell Lines

**FIGURE 4:**
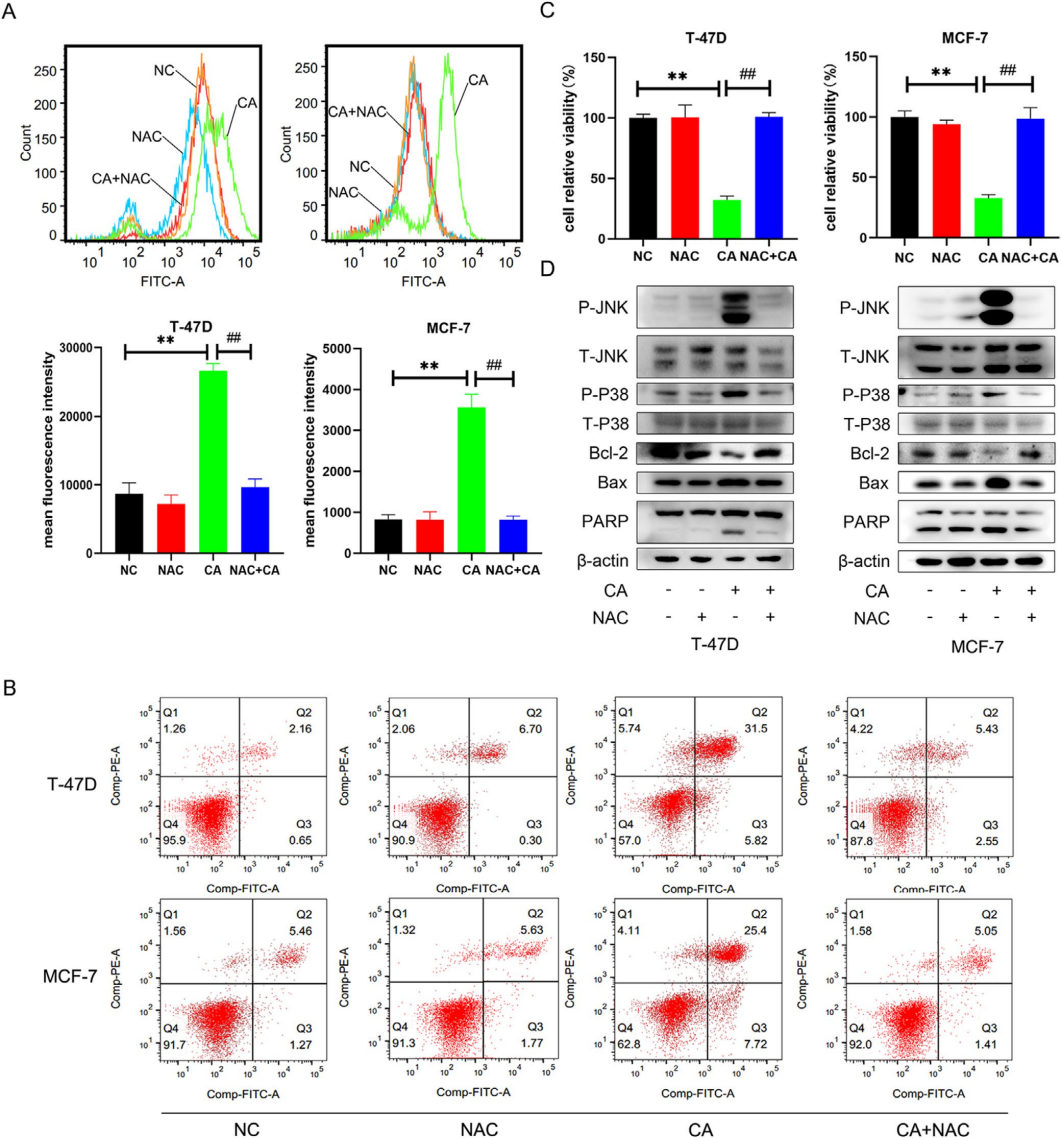
CA induced breast cancer cell apoptosis via the ROS‐MAPK signaling pathway. (A) T‐47D and MCF‐7 cells were treated with the ROS inhibitor NAC (5 mM) and then exposed to CA. The mean fluorescence intensity reflected the level of ROS. (B) Apoptosis rate was evaluated by annexin V and PI staining. (C) Cell viability was determined by MTT assay. (D) Protein expression levels were determined by western blotting. Data presented as mean ± SD of three independent experiments. *p < 0.05, **p < 0.01 versus NC group; ^#^ *p* < 0.05, ^##^ *p* < 0.01 versus CA group.

CA has been reported to induce the generation of intracellular ROS in colon cancer [14]. We therefore examined the level of intracellular ROS in breast cancer cells. T‐47D and MCF‐7 cells were treated with CA 30 and 50 μM for 2 h to generate ROS. ROS levels were detected by flow cytometry following staining with DCF‐DA (Figure 4A). The mean fluorescence intensities of T‐47D cells treated without or with CA were 8668.67 ± 1626.06 and 26,635.33 ± 1041.82, respectively, and the mean fluorescence intensities of MCF‐7 cells were 831.00 ± 113.48 and 3563.33 ± 320.52, respectively. ROS levels were significantly higher in the treatment group compared with the control group. We then abrogated the effect of CA‐induced ROS using the antioxidant NAC (5 mM). The resulting mean fluorescence intensities of T‐47D and MCF‐7 cells were 9636.67 ± 1209.76 and 818 ± 92.59, respectively (Figure 4A). These results indicated that CA induced cellular oxidative stress in breast cancer cells, which could be blocked by NAC.

### 3.5 Role of ROS in CA‐Induced Apoptosis in MCF‐7 and T‐47D Cells Was Mediated via Activation of JNK and p38

To determine if CA regulated cell apoptosis by inducing ROS, we incubated breast cancer cell lines with 5 mM NAC before treatment with CA. NAC pre‐treatment significantly increased relative cell activity compared with cells without NAC pre‐treatment (Figure 4C). Furthermore, pre‐treatment with NAC avoided CA‐induced cell apoptosis. The apoptosis rates in T‐47D and MCF‐7 cells treated with CA were 41.25 ± 4.51 and 36.98 ± 4.99, respectively, and the rates following NAC pre‐treatment were 6.91 ± 0.93 and 5.69 ± 0.79, respectively (Figure 4B). We also examined the related protein pathway, and showed that inhibition by NAC blocked the ROS‐induced phosphorylation of JNK and p38. Pretreatment with NAC averted CA‐induced high expression of Bax and cleavage of PARP in breast cancer cells (Figure 4D). These results showed that CA‐induced ROS generation promoted cell apoptosis by regulating the phosphorylation of JNK and p38.

### 3.6 Anti‐Tumor Effect of CA Assessed by ^99m^Tc‐CN5DG Imaging

**FIGURE 5:**
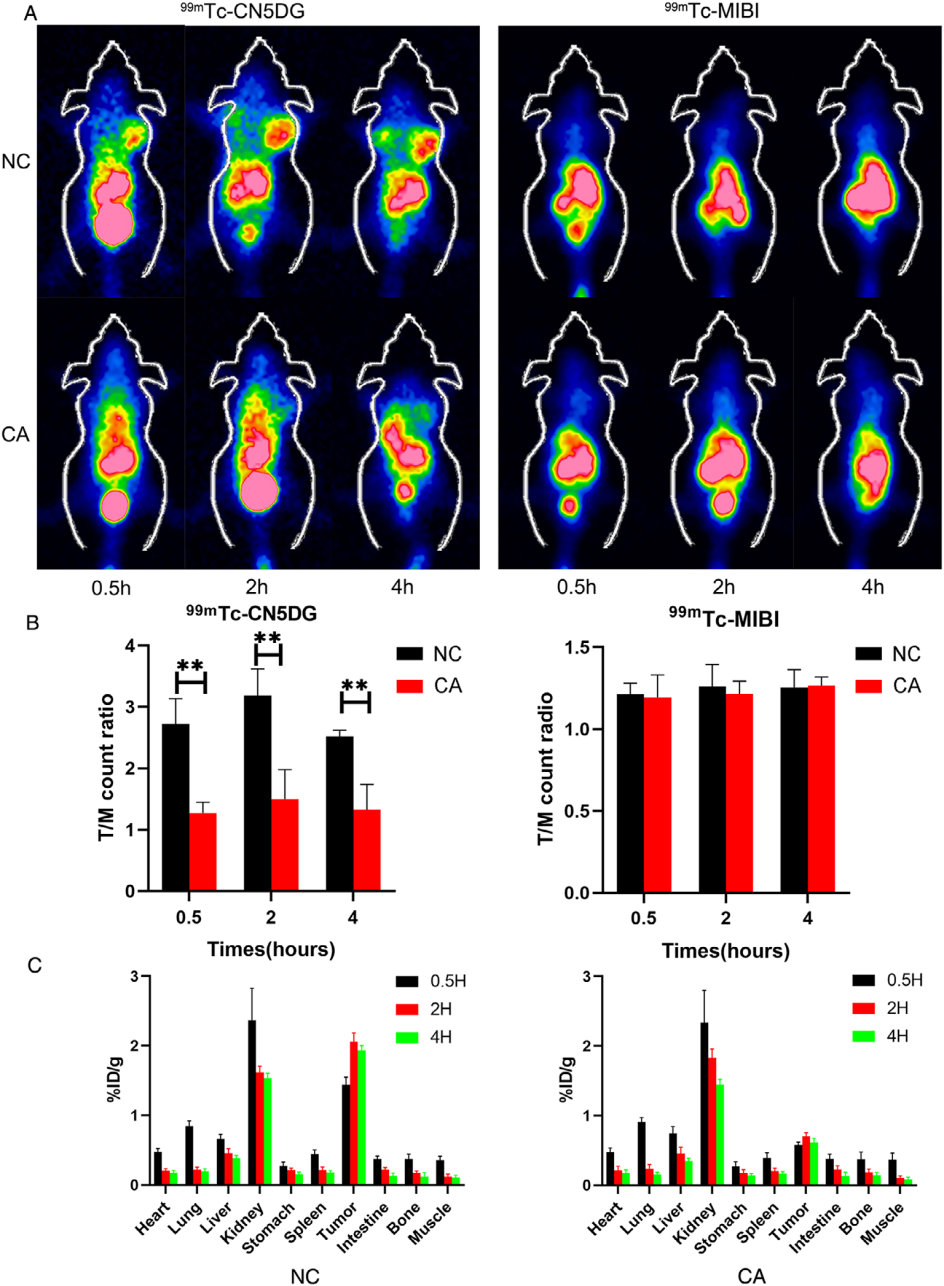
^99m^Tc‐CN5DG and 9^9m^Tc‐MIBI imaging in control and CA‐treated mice. (A) Representative coronal SPECT images with ^99m^Tc‐CN5DG and ^99m^Tc‐MIBI in control and CA‐treated groups. (B) Radioactivity count ratio of tumor versus contralateral muscle. (C) Biodistribution of ^99m^Tc‐CN5DG in control and CA‐treated mice. Data presented as mean ± SD of three independent experiments. *p < 0.05, **p < 0.01 versus NC group.

^99m^Tc‐MIBI is a lipophilic cation that accumulates in mitochondria. ^99m^Tc‐MIBI is also used as a tumor‐imaging agent for the early diagnosis of tumors and for assessing the response to chemotherapy in breast cancer [15, 16]. CN5DG is a glucose analogue that can be labeled with ^99m^Tc, and xenobiotic metabolism levels can be assessed through changes in ^99m^Tc‐CN5DG uptake. We evaluated the anti‐tumor efficacy of CA by ^99m^Tc‐CN5DG and ^99m^Tc‐MIBI imaging. ^99m^Tc‐CN5DG and ^99m^Tc‐MIBI had a radiochemical purity > 95% (Figure S1A,B). The results of SPECT imaging with ^99m^Tc‐CN5DG differed between nude mice treated with or without CA (Figure 5A,B). The T/M ratios in the control group were 2.71 ± 0.42, 3.18 ± 0.42, and 2.52 ± 0.10 at 0.5, 2, and 4 h, respectively, compared with 1.27 ± 0.17, 1.5 ± 0.48, and 1.36 ± 0.34, respectively, in the CA group. As a non‐specific tumor‐targeting imaging agent, ^99m^Tc‐MIBI imaging did not reflect the metabolic changes in the tumor with or without CA treatment. No significant tumor imaging was detected in MCF‐7 xenografts by SPECT. The T/M ratios without CA treatment were 1.21 ± 0.67, 1.26 ± 0.14, and 1.25 ± 0.11 at 0.5, 2, and 4 h, respectively, and the T/M ratios with CA treatment were 1.19 ± 0.14, 1.21 ± 0.77, and 1.26 ± 0.05, respectively. We compared the T/M ratios for CN5DG and MIBI in MCF tumors before and after treatment (Figure S2A,B). These results indicated that ^99m^Tc‐CN5DG could be an effective imaging agent for tracing breast cancer cells and evaluating CA‐induced tumor apoptosis using SPECT.

### 3.7 Biodistribution

To determine the accuracy of SPECT imaging, we estimated the biodistribution of ^99m^Tc‐CN5DG in nude mouse MCF‐7 xenografts with or without CA at different times (Figure 5C). MCF‐7 xenografts showed high accumulation of ^99m^Tc‐CN5DG 0.5 h after injection (2.056 ± 0.13%ID/g), and this then decreased at 2 h (1.78 ± 0.10%ID/g) in the control group. MCF‐7 xenografts in the treatment group also showed a high uptake peak at 0.5 h after injection (0.70 ± 0.05%ID/g), but this was markedly lower than that in the control group and declined at 2 h (0.61 ± 0.06%ID/g). There were no significant differences between the two groups in the other organs evaluated. Higher values were measured in two kidneys at 0.5 and 2 h, but this may have been due to the ^99m^Tc‐CN5DG tracer being rapidly excreted by the urinary system.

### 3.8 CA Suppressed Growth of Breast Cancer Xenografts in Nude Mice

**FIGURE 6:**
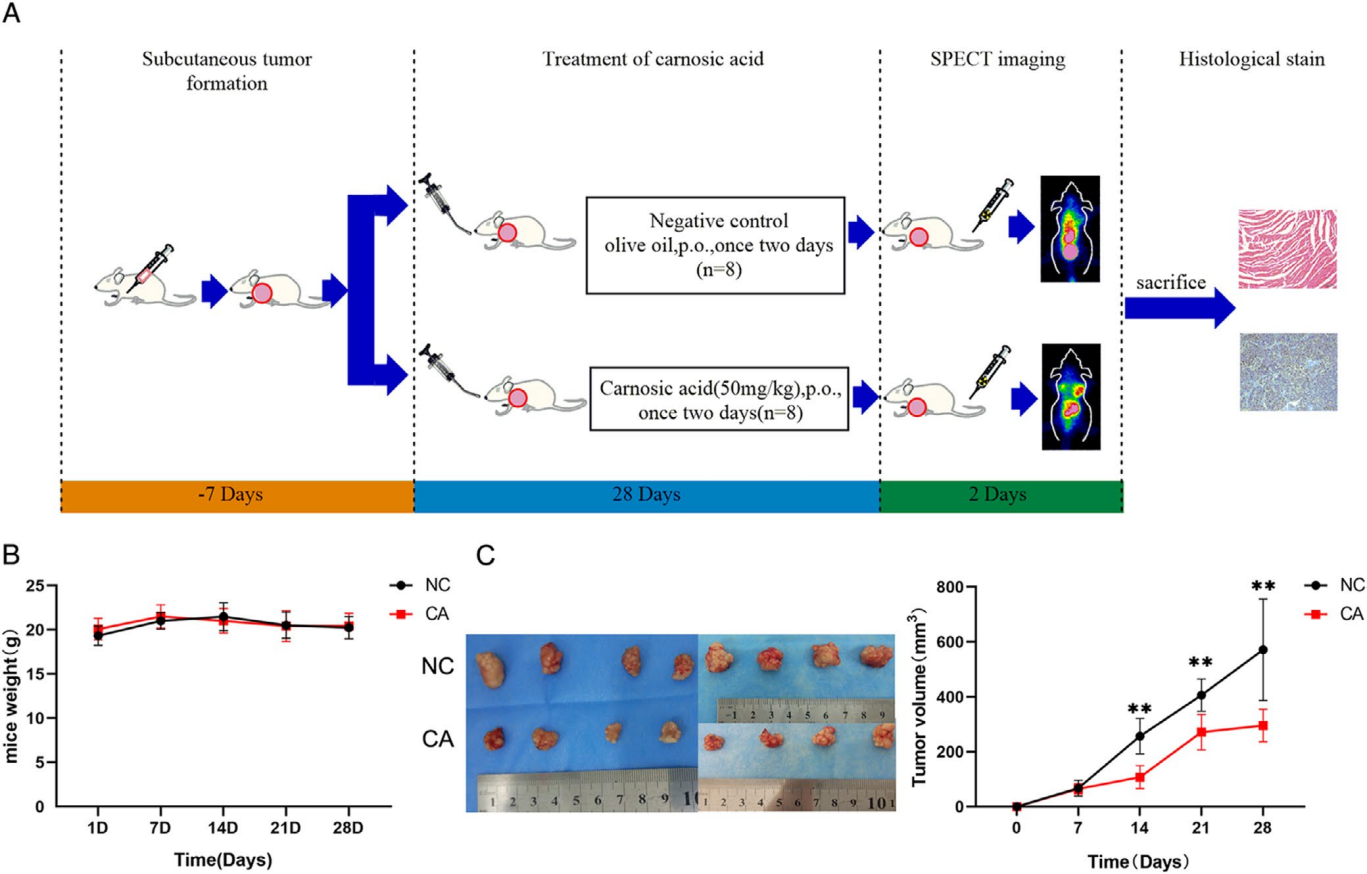
CA suppressed xenograft growth in nude mice. (A) Flow diagram of the experiment. (B) Control and CA 50 mg/kg groups were treated for 4 weeks via oral gavage and body weight was measured every week. (C) Tumor size was recorded every 7 days. After the experiments, the mice were sacrificed by cervical dislocation and the tumors were removed. Data presented as mean ± SD of three independent experiments. *p < 0.05, **p < 0.01 versus NC group.

The animal experiment procedure is shown in Figure 6A. Control mice were treated with vehicle (olive oil) and the treatment group received CA 50 mg/kg once every 2 days by oral gavage. There was no significant difference in body weight between the control and treatment groups (Figure 6B). There was no distinct difference in tumor volume between the two groups in the first 2 weeks. At the beginning of Day 14, tumor volume increased more slowly in the treatment group compared with the control group. At the end of Week 4, the tumor volumes in the control and treatment groups were 571.75 ± 184.44 and 295.76 ± 59.45 mm^3^, respectively (Figure 6C). These findings indicated that CA could suppress breast tumor growth in vivo.

**FIGURE 7:**
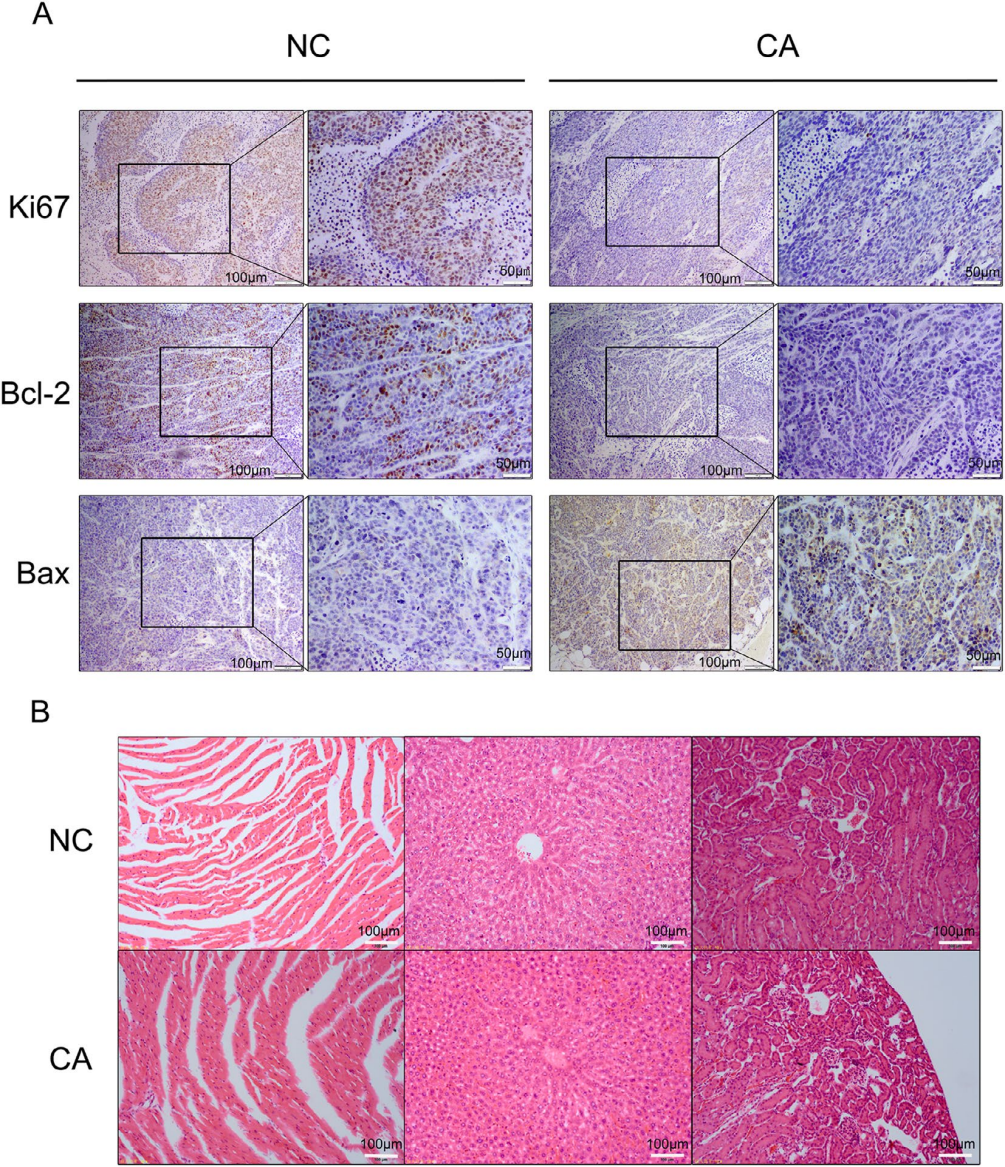
Anti‐tumor effect and organ toxicity of CA in vivo. (A) Tumors were obtained from control and CA‐treated mice and expression levels of Ki67, Bcl‐2, and Bax were determined. (B) CA‐induced toxicity in the heart, liver, and kidney was assessed by HE staining.

CA‐induced tumor apoptosis and growth inhibition in vivo were assessed by immunohistochemistry and spleen index(SI). CA treatment markedly suppressed Ki67 and Bcl‐2 expression levels and increased Bax levels in the tumors compared with the control group (Figure 7A). CA‐induced organ toxicity in vivo assessed by HE staining showed no obvious morphological changes in the vital organs (Figure 7B). The spleen index of the control and treat groups were 5.88 ± 0.87 and 5.82 ± 0.92, respectively (Figure S3). There was no obvious difference between the two groups. These findings indicated that CA could accelerate tumor apoptosis by up‐regulating Bax expression and down‐regulating Ki67 and Bcl‐2 in vivo. Furthermore, CA did not injure vital organs.

## 4 Discussion

Breast cancer is expected to account for 19.5% of all new cancers in women in China [17]. Endocrine therapy is still the main treatment strategy for ER+ breast cancer. Although chemotherapy is an essential treatment for endocrine therapy‐resistant patients, it has several side effects, highlighting the need to identify new drugs with fewer side effects and a defined pharmacological mechanism [18, 19]. CA is a rosemary extract that has demonstrated anti‐tumor activity in various tumors [20, 21, 22]; however, its exact anti‐tumor mechanism and effects are unclear. In the present study, we showed that CA had excellent anti‐tumor activity with no obvious side effects in a mouse breast cancer xenograft model. The results also revealed that ROS‐mediated p38 and JNK phosphorylation were involved in CA‐induced breast cancer apoptosis. Furthermore, ^99m^Tc‐CN5DG imaging could be used to evaluate the anti‐tumor effect induced by CA.

The current study showed that CA decreased the viability and proliferation of breast cancer cells. Although CA showed some cytotoxicity in mammary epithelial cells, its IC50 value was higher in normal than in breast cancer cells. We measured the apoptosis rates of cancer cells treated with CA at different time points, which revealed that CA induced cancer cell apoptosis after 6 h. We also examined the expression levels of proteins in apoptosis pathways. Expression levels of Bax and cleaved PARP were significantly increased, while Bcl‐2 expression was markedly decreased. These results demonstrated that CA could induce breast cancer apoptosis. The MAPK signaling pathway is a complex network involved in the proliferation, metastasis, invasion, and apoptosis of various cancers [23]. MAPK signaling cascades related to breast cancer include four dominant pathways: the ERK1/2, JNK, p38, and ERK5 pathways. p38 is activated by stress signals, growth factors, inflammatory cytokines, UV, heat, and osmotic shock [24], leading to cell death. JNK has a wide range of functions in cells and is known to play a crucial role in triggering apoptosis under cellular and environmental stresses [25]. We therefore investigated if JNK and p38 were involved in CA‐induced apoptosis, and found that CA significantly upregulated the phosphorylation levels of JNK and p38 in vitro. We further clarified the mechanism using the JNK and p38 phosphorylation inhibitors SP600125 and BMS582949; however, these inhibitors failed to block CA‐induced apoptosis, indicating the existence of other determinants regulating cancer cell apoptosis via JNK and p38.

Several forms of DNA damage are caused by ROS, which are associated with tumorigenesis and progression [26, 27, 28]; however, contrasting results have shown that ROS can also induce cell apoptosis, which plays an important role in cancer therapeutics [29, 30]. Numerous studies have indicated that natural compounds can induce cancer cell apoptosis and autophagy via ROS generation [31, 32]. CA has been reported to suppress the STAT3 signaling pathway through ROS generation and inhibit the phosphoinositide 3‐kinase/Akt/mTOR signaling pathway in colon cancer and lung cancer [14, 20]. We therefore considered that CA might upregulate the phosphorylation levels of JNK and p38 by generating ROS. The present results demonstrated that CA promoted cancer cell apoptosis and inhibited cancer cell progression, and that these effects could be abrogated by the antioxidant NAC. We confirmed these findings by measuring cellular ROS levels after CA treatment using DCFH‐DA. The results showed that CA increased ROS levels, and that this increase was abolished by NAC pretreatment. We further investigated the MAPK and mitochondrial apoptosis signaling pathways to clarify the mechanism, and showed that CA induced JNK and p38 phosphorylation, and apoptosis‐related proteins were downregulated by NAC pretreatment. These results demonstrated that CA had an anti‐tumor effect and promoted p38 and JNK phosphorylation by enhancing ROS generation.

In addition to the in vitro experiments, CA also demonstrated effective anti‐tumor activity in vivo. CA decreased the tumor volume in nude mice compared with the control group. Meanwhile, Ki‐67, Bcl‐2, and Bax immunostaining was used to evaluate cell proliferation and apoptosis in tumors in nude mice. Ki‐67 and Bcl‐2 expression levels were reduced while Bax expression was enhanced after CA treatment, indicating that CA could suppress tumor proliferation and promote apoptosis in nude mice. The cell experiments were also validated by immunohistochemistry. Organ toxicity caused by CA in vivo was assessed by HE staining, which revealed no morphological changes in the heart, liver, or kidney in either the control group or the CA group. These results suggest that CA might be a candidate agent for the treatment of hormone‐resistant breast cancer in future clinical practice.

Glucose metabolism is significantly enhanced in malignant tumors and is related to tumor proliferation. ^18^F‐FDG is a glucose analogue that is used to visualize cancer cells in patients using PET/computed tomography (CT) [33], and a higher level of ^18^F‐FDG accumulation is thus a major imaging feature of primary tumors on PET/CT. In addition to tumor diagnosis, ^18^F‐FDG can also be used to assess the response to chemotherapy [34]. Previous studies showed that ^18^F‐FDG accumulation was associated with Ki‐67 [35]. ^18^F‐FDG, however, is a positron imaging agent with a relatively high cost. In the current study, we used ^99m^Tc‐CN5DG and ^99m^Tc‐MIBI as tumor‐imaging agents to assess the anti‐tumor effect of CA. ^99m^Tc‐MIBI is a non‐specific single photon imaging agent that is widely used in the clinical management of breast cancer and hyperthyroidism [36, 37]. The current results, however, showed no significant accumulation of ^99m^Tc‐MIBI in MCF‐7 xenografts before or after treatment. This may have been due to the accumulation of ^99m^Tc‐MIBI in the liver and kidney, which could not be detected clearly on SPECT. Other studies have also shown that Bcl‐2 overexpression prevents the uptake of ^99m^Tc‐MIBI in breast cancer [38]. Conversely, SPECT using ^99m^Tc‐CN5DG demonstrated distinct differences between control and CA‐treated MCF‐7 xenografted mice, with significant accumulation of ^99m^Tc‐CN5DG in MCF‐7 xenografts without CA treatment. Prior to CA treatment, MCF‐7 xenografts consistently showed a higher T/M ratio with ^99m^Tc‐CN5DG than with ^99m^Tc‐MIBI at all time points. The accumulation of ^99m^Tc‐CN5DG decreased after CA treatment in MCF‐7 xenografts, and the T/M ratios of MCF‐7 xenografts were similar with ^99m^Tc‐CN5DG and ^99m^Tc‐MIBI. These results were consistent with the observed glucose uptake by breast cancer cell lines in vitro. These findings indicated that ^99m^Tc‐CN5DG could reflect tumor metabolism in MCF‐7 xenografts, and could be used to assess the anti‐tumor effect of CA.

This study had several potential limitations. The study focused on the induction of ROS by CA, which in turn activates the phosphorylation of JNK and p38 in the MAPK pathway, thereby promoting tumor cell apoptosis; however, we did not measure phosphorylation levels of ERK, another protein involved in the MAPK pathway. Second, the production of ROS triggered by CA in animal experiments is likely to cause significant harm to non‐tumor tissues. We examined the effects of CA on the morphology of important non‐tumor tissues, such as the heart, liver, brain, and kidneys, using HE staining, but did not assess their functions. Further experiments are therefore needed to determine if CA damages the functions of non‐tumor organs, such as transaminase, creatine kinase, and blood creatinine levels. Third, although CA had an obvious anti‐tumor effect, a high concentration was required in vivo. We suggest that CA may lack specific tumor targeting and may have poor bioavailability in animal models. Previous research has shown that CA‐loaded chitosan nanoparticles can prevent liver cancer by affecting the progression of inflammation at a relatively low concentration [39], suggesting that CA may be modified using a nanoplatform and a membrane receptor ligand for co‐delivery into tumors. In addition, this work indicated that CA was an attractive anti‐tumor drug candidate.

## 5 Conclusions

In summary, this study demonstrated that CA can promote breast cancer apoptosis and suppress tumor growth by activating JNK and p38 phosphorylation via ROS generation, both in vitro and in vivo. CA may thus be a potential effective anti‐tumor agent in patients with breast cancer. CA may require further chemical modification, however, to enhance its tumor distribution, which is crucial for its application in clinical translation.

## Author Contributions

**Xinyu Wang:** writing – original draft, methodology. **Peng Xu:** methodology, writing – original draft. **Sha Luan:** methodology, writing – original draft. **Yuying Jiao:** data curation. **Yue Gao:** data curation. **Changjiu Zhao:** conceptualization, funding acquisition. **Peng Fu:** conceptualization, funding acquisition.

## Funding

This study was supported partly by the National Natural Science Foundation of China (grant number 81671714), Scientific Research and Innovation Foundation of 1st Hospital of Harbin Medical University (grant number 2018Y003) and Harbin Medical University Youth Fund (grant number 2023‐KYYWF‐0232).

## Ethics Statement

This study was approved by the Ethics Committee of The First Affiliated Hospital of Harbin Medical University (approval number No. 2017013).

## Conflicts of Interest

The authors declare no conflicts of interest.

## Supporting information


**Figure S1:** The Radiochemical purity of ^99m^Tc‐CN5DG (A) and ^99m^Tc‐MIBI (B).
**Figure S2:** The radioactive count ratio of tumor versus contralateral muscle. (A) ^99m^Tc‐CN5DG versus and ^99m^Tc‐MIBI without CA treatment for 4 weeks. (B) ^99m^Tc‐CN5DG versus and ^99m^Tc‐MIBI with CA treatment for 4 weeks.
**Figure S3:** The spleen index(SI) of control group and CA treatment group.

## Data Availability

The data that support the findings of this study are available from the corresponding author upon reasonable request.
