# Supplementary material for: Carnosic Acid Mediates Production of Reactive Oxygen Species to Regulate Mitogen‐Activated Protein Kinase Pathway Phosphorylation and Induce Apoptosis in Human Breast Cancer Cells
Source: Cancer Med. 2026 Jan 9;15(1):e71446. doi: 10.1002/cam4.71446 (PMC12788981; doi:10.1002/cam4.71446)
Supplement: Supplementary file 1 — Figure S1: The Radiochemical purity of 99mTc‐CN5DG (A) and 99mTc‐MIBI (B). Figure S2: The radioactive count ratio of tumor versus contralateral muscle. (A) 99mTc‐CN5DG versus and 99mTc‐MIBI without CA treatment for 4 weeks. (B) 99mTc‐CN5DG versus and 99mTc‐MIBI with CA treatment for 4 weeks. Figure S3: The spleen index(SI) of control group and CA treatment group. [file CAM4-15-e71446-s001.doc]

**Supplement Figure 1A**

|  | Retention time | Peak Area% | Peak Area |
| --- | --- | --- | --- |
| 1 | 0.017 | 0.1823 | 11877 |
| 2 | 0.066 | 0.4781 | 31149 |
| 3 | 0.109 | 0.1993 | 12985 |
| 4 | 0.523 | 99.06 | 6454477 |
| 5 | 0.951 | 0.07868 | 5127 |
| Total |  | 100 | 6515615 |

**Supplement Figure 1B**

|  | Retention time | Peak Area% | Peak Area |
| --- | --- | --- | --- |
| 1 | 0.258 | 2.051 | 107784 |
| 2 | 0.503 | 97.68 | 5134003 |
| 3 | 0.920 | 0.2676 | 14062 |
| Total |  | 100 | 5255849 |

**Supplement Figure 1.** The Radiochemical purity of ^99m^Tc-CN5DG (A) and ^99m^Tc-MIBI (B).

**Supplement Figure 2**

**
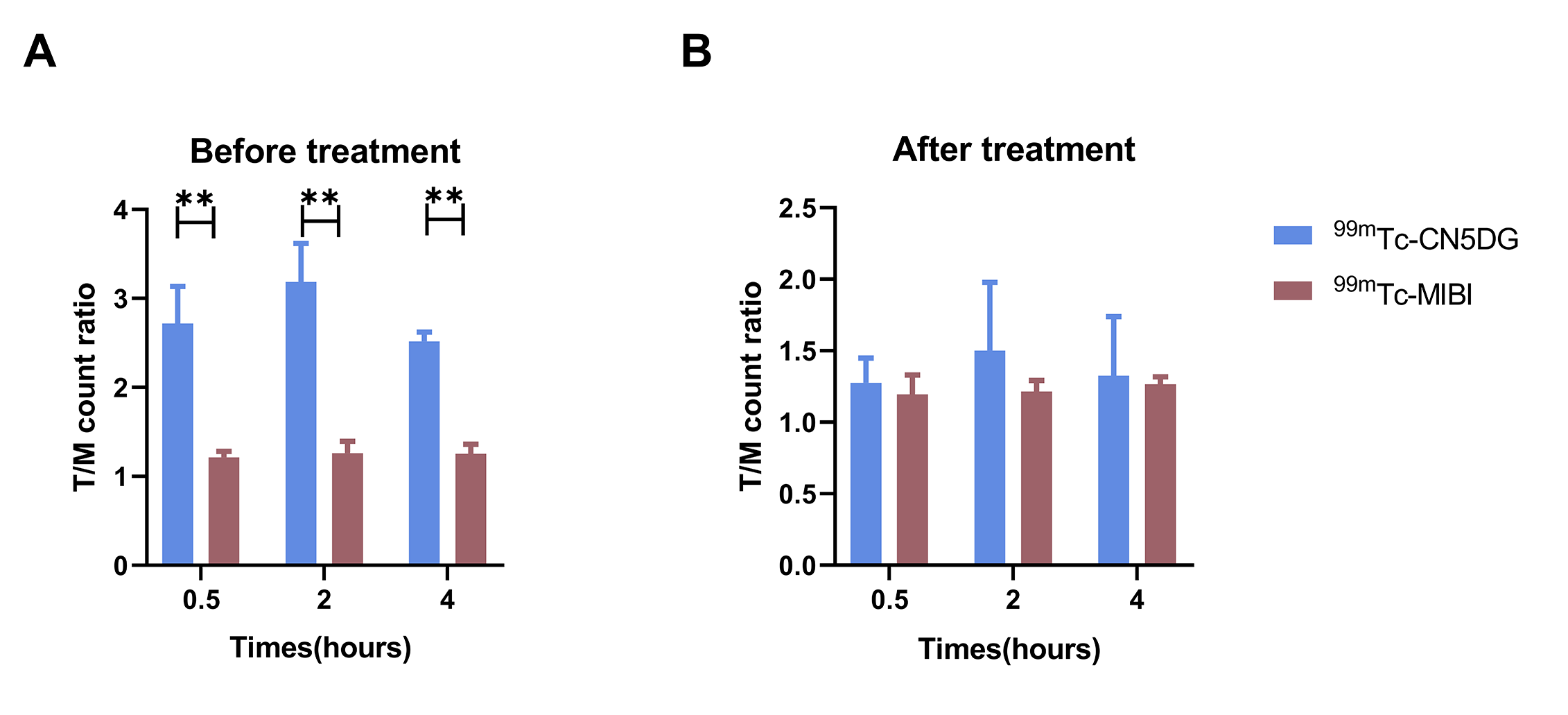
**

**Supplement Figure 2.** The radioactive count ratio of tumor versus contralateral muscle. (A) ^99m^Tc-CN5DG versus and ^99m^Tc-MIBI without CA treatment for four weeks. (B) ^99m^Tc-CN5DG versus and ^99m^Tc-MIBI with CA treatment for four weeks.

**Supplement Figure 3**

**
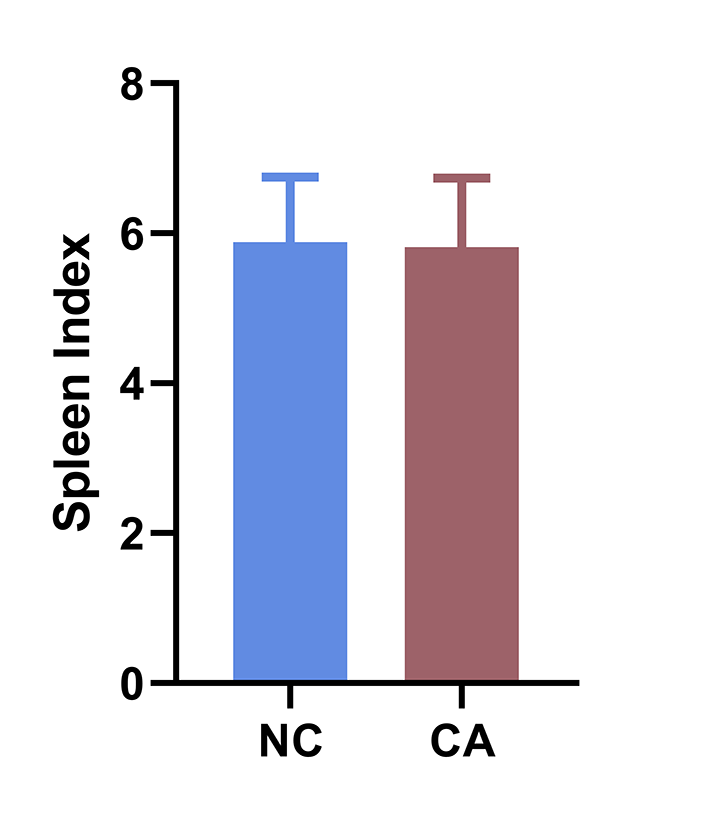
**

**Supplement Figure 3.** The spleen index(SI) of control group and CA treatment group.
